# Alice in Wonderland Syndrome in a Child Following Epstein-Barr Virus Infection: A Case Report

**DOI:** 10.7759/cureus.86532

**Published:** 2025-06-22

**Authors:** Ali Z Ansari, Sabrina L Smith, Andrew S Anderson, Abdul Ahad Siddiqi, Sahar Hafeez

**Affiliations:** 1 Department of Pathology and Laboratory Medicine, William Carey University College of Osteopathic Medicine, Hattiesburg, USA; 2 Department of Neurology, Merit Health Wesley, Hattiesburg, USA; 3 Department of Family Medicine, William Carey University College of Osteopathic Medicine, Hattiesburg, USA; 4 Department of Internal Medicine, William Carey University College of Osteopathic Medicine, Hattiesburg, USA; 5 Department of Family Medicine, William Carey University School of Natural and Behavioral Sciences, Hattiesburg, USA

**Keywords:** alice in wonderland syndrome, body image distortion, derealization, epstein-barr virus, macropsia, micropsia, perceptual distortion, post-infectious neuropsychiatric syndrome, transient perceptual disorder, visual illusions

## Abstract

Alice in Wonderland syndrome (AIWS) is a rare and often underrecognized neuropsychiatric phenomenon characterized by transient episodes of visual and somatosensory perceptual distortions, including micropsia, macropsia, altered body image, derealization, and disrupted perception of time. It most commonly affects children and adolescents and can be triggered by various conditions, including migraine, epilepsy, medications, and infections. We present the case of a 10-year-old previously healthy girl who developed intermittent episodes of micropsia, time distortion, and depersonalization shortly after recovering from a self-limited febrile illness. Her history and symptoms were classic for AIWS, and serologic testing confirmed an acute Epstein-Barr virus (EBV) infection, with positive viral capsid antigen (VCA) IgM and IgG and negative EBNA-1 IgG, indicating a primary EBV infection. Extensive diagnostic workup, including basic metabolic panel, inflammatory markers, autoimmune screening, neuroimaging (MRI), and electroencephalography (EEG), revealed no abnormalities, effectively ruling out structural, epileptic, or metabolic causes. The patient retained full orientation and insight throughout the episodes, and her symptoms gradually resolved over the course of four weeks without pharmacologic intervention. Supportive care, patient and family reassurance, and close outpatient follow-up were sufficient for full recovery. This case reinforces the strong association between AIWS and post-infectious states, particularly EBV, and emphasizes the importance of early recognition and appropriate evaluation to avoid unnecessary interventions. Increased clinician awareness of AIWS is crucial in guiding timely diagnosis, alleviating anxiety, and ensuring optimal management in affected pediatric patients.

## Introduction

Alice in Wonderland syndrome (AIWS) is a rare and distinctive neuropsychiatric condition characterized by transient episodes of perceptual distortion involving visual processing, somatosensory integration, and temporal awareness [1]. The syndrome takes its name from Lewis Carroll’s 1865 novel *Alice’s Adventures in Wonderland*, in which the main character famously experiences dramatic changes in size and a warped sense of reality, phenomena that closely mirror the lived experiences of individuals with AIWS. The term was first formally introduced in 1955 by British psychiatrist Dr. John Todd, who described a constellation of symptoms - most notably micropsia (objects appearing unusually small), macropsia (objects appearing disproportionately large), and altered time perception - which he linked to migraine auras and potential dysfunction in the temporal lobes [2]. While AIWS is infrequently diagnosed, the true incidence may be underestimated due to lack of awareness among clinicians and the difficulty patients, particularly children, may have in describing these unusual sensory changes.

AIWS primarily affects children and adolescents, though cases have been reported in adults as well. Pediatric presentations are particularly noteworthy, as they often occur in the absence of structural brain abnormalities or chronic neurologic disease [3]. Children with AIWS may describe the environment as surreal or dream-like, sometimes feeling detached from their own bodies or surroundings, a sensation known as derealization [4]. In addition to micropsia and macropsia, symptoms may include metamorphopsia (distorted shape perception), body schema disturbances (e.g., perceiving limbs as too large or small), and a disrupted perception of time, such as feeling that time is moving too quickly or too slowly. These episodes are usually episodic and brief, lasting from several minutes to a few hours but can recur over days or weeks [5]. Diagnosing AIWS in children can be challenging, as the unusual and highly subjective nature of symptoms may lead to initial misdiagnosis as a psychiatric disorder, malingering, or anxiety [6]. A thorough history is critical to identifying the condition, and parental observations often provide key insight, especially when children struggle to verbalize their experiences.

The pathophysiology of AIWS is still not completely understood, but current evidence suggests that it is a functional syndrome involving transient disruption in specific areas of the brain - particularly the parieto-occipital cortex, temporoparietal junction, and visual association areas [2]. These regions are integral to spatial orientation, visual processing, and multisensory integration. Functional neuroimaging during symptomatic episodes has shown hypo- or hyperperfusion in these areas, supporting the theory of a dynamic cerebral dysfunction rather than a structural lesion [7]. While migraines are the most frequently reported underlying cause, especially in adults, AIWS has also been associated with temporal lobe epilepsy, encephalitis, traumatic brain injury, brain tumors, psychiatric disorders, and pharmacologic side effects [8]. In children, however, infectious etiologies are emerging as a leading cause, with viruses such as Epstein-Barr virus (EBV) and influenza A implicated in multiple case reports [9,10]. EBV, in particular, is increasingly recognized as a common post-infectious trigger in pediatric AIWS, possibly through transient inflammatory or immune-mediated effects on the central nervous system [9]. Once structural, epileptic, and psychiatric causes have been excluded, management is largely supportive, focusing on reassurance, symptomatic care, and monitoring for recurrence.

## Case presentation

A 10-year-old previously healthy girl with no known past medical, neurological, or psychiatric history presented to the pediatric neurology service for evaluation of new-onset episodic perceptual disturbances. Her symptoms were characterized by transient visual distortions, altered body perception, and significant time dysperception. The patient had been in her usual state of health until approximately four weeks prior to presentation, when she developed a self-limited illness characterized by low-grade fever, fatigue, sore throat, and mild frontal headache. These symptoms lasted approximately five days and resolved spontaneously without medical intervention. No antibiotics, corticosteroids, or other systemic medications were administered during the illness. She remained afebrile after the third day and continued to engage in daily activities, albeit with reduced energy. No clinician was consulted at the time; the illness was presumed to be viral in origin. There was no associated rash, photophobia, vomiting, diarrhea, altered sensorium, or loss of consciousness. However, her parents noted transient, bilateral, non-tender cervical lymphadenopathy, which gradually resolved by the end of the illness.

Approximately 10 days following resolution of the febrile illness, the patient began to experience sudden-onset episodes of visual and somatosensory distortion. The first episode occurred while she was reading at home, during which she reported that the pages of her book appeared to expand and the words seemed to stretch unnaturally across the paper. She also perceived that her limbs were growing unusually long (macrosomatognosia), while surrounding furniture appeared to shrink in size (micropsia). These visual distortions were accompanied by a sensation that time had slowed down and a feeling of detachment from her surroundings, which she described as “dream-like” or “unreal.” She remained fully conscious, oriented, and communicative throughout each episode and was able to describe the experience in real time and recall the details afterward with clarity. These episodes typically lasted 10-20 minutes and occurred several times per day over the subsequent two weeks.

During this time, the patient did not report any associated headaches, nausea, vomiting, diplopia, auditory disturbances, limb weakness, color vision abnormalities, or hallucinations. There was no loss of consciousness or signs suggestive of seizure activity, such as automatisms, tonic-clonic movements, urinary incontinence, or postictal confusion. The episodes were frightening to her, but she consistently maintained insight and was able to differentiate between her perceptions and reality. There was no history of trauma, recent travel, tick bites, medication or supplement use, or exposure to recreational drugs or environmental toxins. Her appetite, sleep patterns, and energy levels remained stable outside of the episodes. There were no behavioral changes, academic difficulties, or social withdrawal reported by caregivers or teachers. The family history was non-contributory. There was no known history of epilepsy, migraines, autoimmune disease, psychiatric illness, or metabolic disorders in first- or second-degree relatives. She lived with both biological parents and two younger siblings in a stable household, had no pets, and was up-to-date on all vaccinations. The family reported no international or domestic travel in the previous six months.

On physical examination, the patient appeared well-nourished and in no acute distress. The patient's vital signs were within normal limits for her age. Her axillary temperature was 36.8°C, heart rate was 88 beats per minute, respiratory rate was 18 breaths per minute, blood pressure measured 102/64 mmHg, and oxygen saturation was 99% on room air. She was afebrile at the time of examination and showed no signs of hemodynamic instability, respiratory distress, or dehydration. Her growth parameters were appropriate for age, with both height and weight tracking along the 40th percentile according to the Centers for Disease Control and Prevention (CDC) pediatric growth charts. Capillary refill time was less than two seconds, and peripheral pulses were strong and symmetric throughout. General examination was unremarkable except for residual, bilateral, non-tender cervical lymphadenopathy measuring approximately 1 cm in diameter.

Neurological examination was comprehensive and age-appropriate. She was alert and cooperative, with intact orientation to time, place, and person. Her language was fluent, with appropriate vocabulary and syntax. Attention, memory, and fund of knowledge were age-consistent. Cranial nerve examination was normal, including visual fields, extraocular movements, pupillary responses, and fundoscopic evaluation, which revealed no papilledema or retinal abnormalities. Motor strength was 5/5 bilaterally, with normal tone and bulk. Deep tendon reflexes were symmetric and brisk. Sensory examination was intact to light touch, pinprick, and proprioception. Cerebellar function, assessed through finger-to-nose and heel-to-shin testing, was normal. Gait was steady, with intact tandem walking and no extrapyramidal signs.

Given the nature and timing of her symptoms, the working diagnosis was AIWS, characterized by micropsia, macrosomatognosia, time distortion, and derealization. A broad differential diagnosis was considered, including migraine aura without headache, focal seizures (particularly temporal or occipital lobe epilepsy), post-infectious encephalopathy, toxic-metabolic encephalopathy, autoimmune encephalitis (e.g., anti-NMDA (N-methyl-D-aspartate) receptor encephalitis), and psychiatric conditions such as early-onset schizophrenia or depersonalization-derealization disorder. There was no evidence of traumatic injury, psychosis, or intoxication. Initial laboratory investigations were aimed at evaluating for infectious, autoimmune, metabolic, and toxic etiologies (Table 1).

**Table 1:** Laboratory test results demonstrated unremarkable hematologic, metabolic, hepatic, thyroid, autoimmune, and inflammatory profiles. Infectious disease screening and toxicology results were negative, with the exception of serologic evidence of a primary EBV infection, indicated by positive VCA IgM and IgG with negative EBNA-1 IgG. EBV: Epstein-Barr virus; VCA: viral capsid antigen; EBNA: Epstein-Barr nuclear antigen 1; CMV: cytomegalovirus; NMDA: N-methyl-D-aspartate.

Test	Patient's value	Reference Range
White blood cell count	7.4 ×10³/µL	4.5-13.5 ×10³/µL
Hemoglobin	13.1 g/dL	11.5-15.5 g/dL
Platelet count	256 ×10³/µL	150-450 ×10³/µL
Erythrocyte sedimentation rate	8 mm/h	<20 mm/h
C-reactive protein	<0.5 mg/dL	<1.0 mg/dL
Aspartate transaminase	32 U/L	10-40 U/L
Alanine transaminase	29 U/L	10-45 U/L
Thyroid stimulating hormone	2.1 µIU/mL	0.5-4.3 µIU/mL
EBV VCA IgM	Positive	Negative
EBV VCA IgG	Positive	Positive or negative
EBNA-1 IgG	Negative	Negative
CMV IgM	Negative	Negative
CMV IgG	Negative	Negative
Antinuclear Antibody	Negative	Negative
Anti-NMDA receptor antibodies	Negative	Negative
Toxicology screen (urine)	Negative	Negative
Sodium	138 mmol/L	135–145 mmol/L
Potassium	4.3 mmol/L	3.5–5.0 mmol/L
Chloride	102 mmol/L	98–107 mmol/L
Bicarbonate	24 mmol/L	22–29 mmol/L
Blood urea nitrogen	10 mg/dL	7–18 mg/dL
Creatinine	0.6 mg/dL	0.4–1.0 mg/dL
Glucose (random)	91 mg/dL	70–110 mg/dL
Calcium	9.4 mg/dL	8.5–10.5 mg/dL

Magnetic resonance imaging (MRI) of the brain with and without contrast revealed normal brain parenchyma, with no structural lesions, demyelination, or signs of increased intracranial pressure. Ventricular size and configuration were normal. An electroencephalogram (EEG) performed during both wakefulness and drowsiness demonstrated age-appropriate background rhythms with no epileptiform activity, focal slowing, or photoparoxysmal response. A pediatric neuropsychology consultation was obtained to assess for psychiatric or cognitive comorbidities. Abbreviated screening revealed no evidence of hallucinations, delusions, attention deficits, or cognitive decline. The patient demonstrated intact insight and reality testing, with no indicators of malingering or factitious disorder. A lumbar puncture was not performed, as the patient exhibited no signs or symptoms of central nervous system infection or meningeal irritation. Her neurological examination was entirely normal, and there were no features suggestive of encephalitis or meningitis such as altered mental status, photophobia, neck stiffness, or focal deficits. Given the absence of red flags and reassuring neuroimaging and EEG findings, invasive testing was deemed unnecessary.

Based on the clinical history, characteristic perceptual phenomena, absence of neurological or psychiatric comorbidity, and laboratory evidence of acute primary EBV infection, a final diagnosis of AIWS secondary to EBV was made. The patient and family were extensively counseled about the benign and typically self-limiting nature of AIWS. Given the absence of seizures, structural lesions, or psychiatric symptoms, no pharmacologic therapy was initiated. Supportive care, reassurance, and routine outpatient neurology follow-up were recommended. The family was advised to maintain a detailed symptom diary to monitor the frequency and pattern of episodes and to implement general wellness measures, including good sleep hygiene, reduction in screen time, and avoidance of sensory overstimulation. At one-month follow-up, the frequency and severity of the episodes had diminished substantially, with milder and shorter events. By the end of the fourth week, all perceptual symptoms had completely resolved. At three months, the patient remained asymptomatic and continued to perform well academically and socially. At six months, there was no recurrence of symptoms, supporting a favorable outcome consistent with the natural history of post-infectious AIWS. A summary of the timing, frequency, duration, and characteristics of the patient’s perceptual episodes throughout the course of illness is provided in Table 2.

**Table 2:** The timing, frequency, duration, and characteristics of the patient’s perceptual episodes following an Epstein-Barr virus infection. N/A: Not applicable; AIWS: Alice in Wonderland syndrome

Timeline	Clinical Phase	Episode Frequency	Typical Duration	Key Perceptual Features	Associated Clinical Features
Week 0	Febrile illness	None	N/A	None	Low-grade fever, fatigue, sore throat, mild frontal headache; no neurologic or systemic red flags
Days 6–10 post-illness	Recovery phase	None	N/A	None	Afebrile; mild fatigue; engaging in daily activities; bilateral cervical lymphadenopathy resolving
Day 10 post-illness onward (Week 2)	Initial onset of AIWS episodes	Several times per day	10–20 minutes	Micropsia, macrosomatognosia, time distortion, derealization (“dream-like” feeling)	Fully conscious, oriented, communicative; able to describe and recall episodes in real time
Week 3	Active symptom period	Several times per day	10–20 minutes	Same as above; visual distortions (stretching text, shrinking furniture), altered time	No headache, nausea, diplopia, hallucinations, weakness, or seizure signs
Week 4	Improvement phase	Several times per week	Brief (<10 minutes)	Diminishing micropsia, rare derealization	No behavioral, cognitive, or functional deficits reported
End of Week 4	Symptom resolution	None	N/A	None	All perceptual symptoms resolved
Month 3 follow-up	Asymptomatic period	None	N/A	None	Doing well academically and socially
Month 6 follow-up	Continued symptom-free state	None	N/A	None	No recurrence; remained developmentally and neurologically normal

## Discussion

The case presented involves a previously healthy 10-year-old girl who developed hallmark features of AIWS-specific intermittent episodes of micropsia, derealization, and temporal distortion - shortly after recovering from a self-limited febrile illness. Serologic testing confirmed a primary EBV infection, consistent with the typical profile of infectious mononucleosis [11]. The striking temporal relationship between the onset of perceptual symptoms and the resolution of her viral illness, combined with a comprehensive but unremarkable diagnostic evaluation, supports the diagnosis of post-infectious AIWS. Importantly, this case illustrates a classic clinical trajectory in which otherwise healthy children may experience transient neuropsychiatric symptoms in the wake of an acute viral illness, and it highlights the diagnostic value of recognizing this unique syndrome early in its course.

In pediatric populations, AIWS is increasingly recognized as a benign condition, though an unusual follow-up symptom of various viral infections. EBV is among the most frequently implicated pathogens, along with other members of the herpesvirus family, including cytomegalovirus (CMV) and human herpesvirus 6 (HHV-6), as well as respiratory viruses like influenza A and B [12]. EBV has been associated with a spectrum of neurologic complications, ranging from encephalitis and cerebellitis to immune-mediated syndromes such as Guillain-Barré syndrome and acute disseminated encephalomyelitis [13]. In the context of our case, the confirmation of acute EBV infection provides a biologically plausible explanation for the transient cortical dysfunction underlying the patient's perceptual disturbances.

While EBV is well known for causing infectious mononucleosis, its neurological manifestations are relatively rare and often subtle. In cases like this one, AIWS is a diagnosis of exclusion that must be approached with a broad differential. The patient underwent an extensive diagnostic workup, including complete blood count, inflammatory markers, comprehensive metabolic panel, autoimmune screening, serologic testing for infectious agents, MRI of the brain, and EEG. These investigations ruled out metabolic imbalances, structural brain lesions, seizure activity, autoimmune encephalitis, and toxic exposures. Of note, the serologic profile - positive EBV VCA IgM and IgG with negative EBNA-1 IgG - confirmed a primary EBV infection [11]. The absence of findings on MRI and EEG further supported a functional, rather than structural or epileptic, cause for the patient's symptoms, helping to solidify the diagnosis of post-infectious AIWS.

The pathophysiological mechanisms of AIWS are not fully understood but are thought to involve transient dysfunction of the temporoparietal-occipital junction, a key integrative hub responsible for processing visual, spatial, and somatosensory information [2]. Functional neuroimaging studies, including positron emission tomography (PET) scans in patients experiencing AIWS, have demonstrated regional hypoperfusion or hypoactivation in the occipital, temporal, and parietal cortices-regions implicated in visual-spatial perception and self-representation [14]. In post-viral AIWS, several mechanisms have been proposed, including cytokine-mediated neuroinflammation, transient autoimmune cross-reactivity, and direct viral effects on neuronal or glial function. EBV's ability to infect B cells and potentially enter the central nervous system may contribute to a reversible disruption of functional neural circuits, without causing overt structural damage [11,13,15]. The self-limited course observed in this patient is consistent with the proposed pathophysiology of post-infectious AIWS, wherein transient immune-mediated or inflammatory changes within the brain disrupt sensory integration without causing lasting structural damage. As these functional perturbations subside over time, likely due to the resolution of neuroinflammation and restoration of normal neurotransmission, the perceptual distortions gradually diminish and disappear.

Clinically, distinguishing AIWS from other neurologic or psychiatric conditions is critical, particularly in pediatric patients, where unusual perceptual experiences may be misattributed to malingering, anxiety, or even psychosis [6]. AIWS must be differentiated from temporal lobe epilepsy, migraine aura, intoxication, psychotic disorders, and dissociative conditions. Our patient exhibited full orientation, intact reality testing, preserved emotional regulation, and age-appropriate insight throughout her illness - all of which are inconsistent with primary psychiatric disorders or seizure activity. Her episodes were episodic, brief, and stereotyped, lacking features such as auras, automatisms, hallucinations, or altered consciousness, which helped exclude epilepsy and migraine. This diagnostic clarity allowed for focused management without unnecessary pharmacologic or psychiatric intervention [16].

Management of AIWS in children, particularly when linked to a recent infection, is typically conservative. The priority is to provide reassurance to both the patient and their caregivers, emphasizing the benign and self-limited nature of the syndrome [17]. In this case, the patient’s symptoms, though disorienting, were not severe enough to interfere with school attendance, daily functioning, or sleep. Supportive care strategies included education about the expected course of illness, validation of the patient’s experiences, and behavioral recommendations such as maintaining a consistent sleep schedule, reducing screen time, and keeping a symptom diary. These approaches empowered the patient and family while minimizing anxiety. Over the subsequent month, her symptoms gradually abated without the need for pharmacologic therapy, and she remained symptom-free at follow-up three months later. This natural resolution further supports the notion that post-infectious AIWS typically follows a benign course.

Although pharmacologic treatment is rarely needed, it may be considered in cases where AIWS symptoms are persistent, functionally impairing, or occur in conjunction with an underlying neurological or psychiatric disorder. Antiepileptic medications may be beneficial in patients with coexisting seizure disorders, while migraine prophylaxis may help in cases with overlapping migraine symptoms [18]. In patients where anxiety or mood disturbances exacerbate perceptual symptoms, selective serotonin reuptake inhibitors (SSRIs) or short-term anxiolytics have been used anecdotally [19,20]. However, none of these interventions were required in our case, reinforcing the importance of symptom monitoring and conservative management in mild, uncomplicated presentations.

This case highlights the crucial role of clinician awareness and diagnostic confidence in managing AIWS. Because the syndrome involves subjective and often difficult-to-describe experiences, especially in younger children, it is frequently misdiagnosed or overlooked. When AIWS is misattributed to psychosis, seizure disorders, or malingering, patients may undergo unnecessary investigations, hospitalizations, or inappropriate psychiatric treatment. A thorough but focused diagnostic strategy that includes detailed history-taking, neurological examination, targeted infectious disease testing, and neuroimaging can typically distinguish AIWS from other serious conditions. Educating frontline providers, including pediatricians, neurologists, and emergency clinicians, about the key features of AIWS is essential for timely recognition and effective reassurance.

## Conclusions

AIWS is a rare but distinct neuropsychiatric condition characterized by transient perceptual distortions, most commonly affecting children and adolescents. This case highlights the importance of recognizing AIWS in the context of recent viral infections - particularly EBV - as a potential trigger. Despite its dramatic presentation, AIWS is typically self-limited and benign, requiring no pharmacologic intervention in most cases. Prompt recognition, appropriate exclusion of more serious neurologic or psychiatric conditions, and reassurance of both the patient and family are key to effective management. As awareness grows, clinicians can better identify and document this underrecognized syndrome, contributing to improved understanding of its pathophysiology, natural history, and outcomes.
